# Extreme specificity in obligate mutualism—A role for competition?

**DOI:** 10.1002/ece3.11628

**Published:** 2024-06-21

**Authors:** Renuka Agarwal, David M. Althoff

**Affiliations:** ^1^ Department of Biology Syracuse University Syracuse New York USA

**Keywords:** community structure, competitive exclusion, guilds, specificity

## Abstract

Obligate mutualisms, reciprocally obligate beneficial interactions, are some of the most important mutualisms on the planet, providing the basis for the evolution of the eukaryotic cell, the formation and persistence of terrestrial ecosystems and the establishment and expansion of coral reefs. In addition, these mutualisms can also lead to the diversification of interacting partner species. Accompanying this diversification is a general pattern of a high degree of specificity among interacting partner species. A survey of obligate mutualisms demonstrates that greater than half of these systems have only one or two mutualist species on each side of the interaction. This is in stark contrast to facultative mutualisms that can have dozens of interacting mutualist species. We posit that the high degree of specificity in obligate mutualisms is driven by competition within obligate mutualist guilds that limits species richness. Competition may be particularly potent in these mutualisms because mutualistic partners are totally dependent on each other's fitness gains, which may fuel interspecific competition. Theory and the limited number of empirical studies testing for the role of competition in determining specificity suggest that competition may be an important force that fuels the high degree of specificity. Further empirical research is needed to dissect the relative roles of trait complementarity, mutualism regulation, and competition among mutualist guild members in determining mutualism specificity at local scales.

## Graphical abstract

The high degree of specificity in obligate mutualisms is driven by competition within obligate mutualist guilds that limits species richness. As mutualistic partners are completely dependent on each other for fitness gains, competition may be particularly potent in these mutualisms.

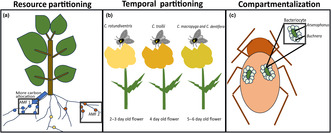

## 1 INTRODUCTION

Mutualism, a reciprocally beneficial species interaction, helps form the foundation of life on earth and can involve vast networks of loosely dependent species to highly specialized and specific pairwise obligate relationships (Boucher et al., 1982; Bronstein, 2015; Douglas, 2010). Mutualistic interactions can occur among very different organisms across the tree of life such as between microbes and animals, fungi and plants, as well as more closely related organisms such a cleaner fish and their hosts, and ants and the aphids they tend (Ayres, 2016; Cheney & Côté, 2005; Clay, 1988; Douglas, 2010; Stadler & Dixon, 2005). In general, most mutualistic interactions are facultative, usually involving a number of species on both sides of the mutualism and exhibit a low level of partner fidelity. For example, many plant species are visited and pollinated by dozens of pollinator species (Wardhaugh, 2015; Waser et al., 1996). At the opposite extreme, two mutualistic species can become completely dependent on one another for their fitness and be locked together evolutionarily for millions of years such as in yuccas and their yucca moth pollinators, senita and senita moth pollinators or figs and their fig wasp pollinators (Cruaud et al., 2012; Fleming & Holland, 1998; Pellmyr & Leebens‐Mack, 1999). One of the challenges in the evolutionary ecology of mutualism is understanding the processes that dictate why a mutualism will move along the continuum of low to high specificity and produce extremely specific and specialized interactions (Chomicki et al., 2020). Here we posit that for obligate mutualism, in particular, the high degree of reciprocity centered on a single resource or service provides the context for competition among mutualist guild members that may influence local species richness of interacting mutualist partners.

Obligate mutualisms represent some of the most striking examples of specificity and specialization in species interactions. Many obligate mutualisms have a one‐to‐one matching of mutualist species (extreme specificity) and exhibit specialized traits that help facilitate the mutualism. For example, in active pollination systems, pollinating insects have specialized morphologies such as the tentacular mouthparts in yucca moths and pollen pockets in fig wasps that are not seen in other pollinating taxa (Pellmyr & Krenn, 2002; Ramírez, 1969). Fungi living in attine ant nests have specialized hyphal sacs that provide nutrients to their ant tenders (Augustin et al., 2013). Similarly, obligate microbes living within the bodies of their hosts have significantly reduced genomes in comparison to free‐living relatives and rely on their hosts to provide needed metabolic products (Boscaro et al., 2017; McCutcheon & Moran, 2012; Wernegreen, 2002; Xiao et al., 2013). These examples highlight how trait evolution goes hand in hand with specialization in obligate mutualisms.

Trait complementarity is thought to improve the ability of mutualists to interact and maximize benefits while also reducing costs of participating in the mutualism. This is likely the reason that we see highly specialized traits evolve in many obligate mutualisms. Established mutualists have already gone through cycles of (co)evolution to maximize benefits and reduce costs and hence have specialized to the traits of their partners. As part of this process, trait complementarity may be so important that any deviation from the optimal trait value will result in reduced mutualistic benefit being offered or gained. Theory suggests that mutualisms may be under strong selection that maintains trait complementarity between interacting partner species (Gomulkiewicz et al., 2003; Johnson & Steiner, 2000; Kiers et al., 2010; Yoder & Nuismer, 2010). For example, the receptive stigmatic surface in yuccas is in a ring that is several millimeters below the top to the pistil and yucca moths must use unique, specialized mouthparts and stereotypical behavior to push the pollen into this cup to pollinate the flowers (Pellmyr, 2003). Although incidental pollination is possible, it is extremely rare in yuccas (Rentsch & Leebens‐Mack, 2014). Thus, it would be difficult for other species to become co‐pollinators without the proper morphology and behavior. Additionally, yucca flowers offer no reward in terms of nectar that would attract other potential pollinators. Thus, the suite of specialized traits for this pollination mutualism serves to limit the opportunities to engage with other potential mutualist species, and may reinforce the high degree of specificity observed in this mutualism.

Although trait evolution in obligate mutualisms may narrow the number of potential partner species due to lack of trait complementarity, it does not necessarily preclude other mutualist species with similar, complementary traits from also interacting. For example, in yucca moths and figs and fig wasps, there are several instances in which two pollinator species use the same plant species at a locality (Leebens‐Mack et al., 1998; Machado et al., 2005; Yang et al., 2015) and leafflowers can have two or three co‐occuring leafflower moths (Kawakita, 2010; Kawakita & Kato, 2006). Chomicki et al. (2020) demonstrate that there are also examples of obligate mutualisms that are also very generalized such as obligate outcrossing plants and their diverse pollinators, A.M.F. (arbuscular mycorrhizal fungi) and most plants, gut microbes and their hosts, fleshy‐fruited plants and their dispersers. In these instances, however, only one mutualist type is obligately dependent on its interacting partners. Thus, it is unlikely that selection for trait complementarity alone can explain the diversity of specificity in obligate mutualisms. Similarly, trait complementarity cannot fully explain multiple mutualist species interacting in facultative mutualisms.

The difference in mutualist community structure between facultative and obligate mutualisms and even within obligate mutualisms with asymmetries in dependency suggests that there may be additional processes that are important in structuring mutualist communities. In any community or guild of organisms that use the same limiting resource, competition for that resource can be a strong force in structuring community size and species persistence (Chesson, 2000; Simberloff & Dayan, 1991; Tilman, 1994, 2004). Theoretical studies focused on mutualism suggest the same pattern (Gosh et al., 2022; Johnson & Bronstein, 2019). Palmer et al. (2003) were among the first to review empirical studies on competition and coexistence in mutualist communities, specifically plant–pollinator and ant–plant mutualisms. They concluded that there is ample opportunity for competition to be important among mutualist guild members and identified mechanisms that would allow the coexistence of guild members. Interestingly, however, there was little empirical data examining competition among mutualists. Almost a decade later, Jones et al. (2012) presented a series of graphical framework models and again reviewed empirical evidence from published studies to detail the ways in which both intra‐ and interspecific competition can be important in mutualism for influencing the evolution of rewards, cheating, specialization, and partner specificity. They suggest that competition for mutualistic partners may serve to drive specialization and hence specificity in many types of mutualism. Again, they detailed that there were relatively few studies focusing on competition and its role in driving mutualism dynamics and community structure and that competition may be an important but largely overlooked factor in mutualism.

Here, we argue that the extreme specificity seen in many obligate mutualisms may be partly or mainly explained by competition among mutualists for mutualistic goods or services offered by partner species. Theoretical studies predict that as the pool of species involved in a mutualism at the local scale increases, species start competing more intensely for shared resources, potentially limiting mutualist guild size (Ferrière et al., 2007; Jones et al., 2012; Palmer et al., 2003). We focus on obligate mutualisms because they have produced striking examples of trait specialization among interacting species and this process has been assumed to be important in driving specificity. Previous authors have drawn attention to the potential role that competition may have in structuring mutualist communities in general (Johnson & Amarasekare, 2013; Jones et al., 2012; Palmer et al., 2003) and Chomicki et al. (2020) highlighted the role of dependency in asymmetry in mutualistic interactions that may also influence community structure. Here, we focus specifically on obligate mutualisms to assess the potential role of competition in generating extreme specificity in these interactions. Obligate mutualisms have been the basis for understanding the evolutionary ecology of mutualism, cooperation, and cheating, as well as taxa diversification. As such, these systems represent a logical starting point for highlighting the role of competition in structuring mutualist communities. We define mutualism as obligate if one of the mutualists requires its partner species to survive and reproduce. This would include mutualisms designated as obligate generalists and obligate specialists by Chomicki et al. (2020).

To address this idea, we answer the following questions concerning specificity in obligate mutualisms:

1. When focusing at the local scale, do obligate mutualisms across a diverse array of taxonomic groups usually have a small number of interacting species on each side of the interaction?
2. Based on this finding, is there empirical evidence that competitive exclusion among mutualist guild members may lead to extreme specificity in obligate mutualistic partners?
3. Conversely, for obligate mutualisms in which there are multiple guild members coexisting at a local scale, is there evidence for mechanisms that reduce competition and promote coexistence among competing guild members?

### 1.1 Q1. Do obligate mutualisms across a diverse array of taxonomic groups usually have a small number of interacting species on each side of the interaction?

**FIGURE 1:**
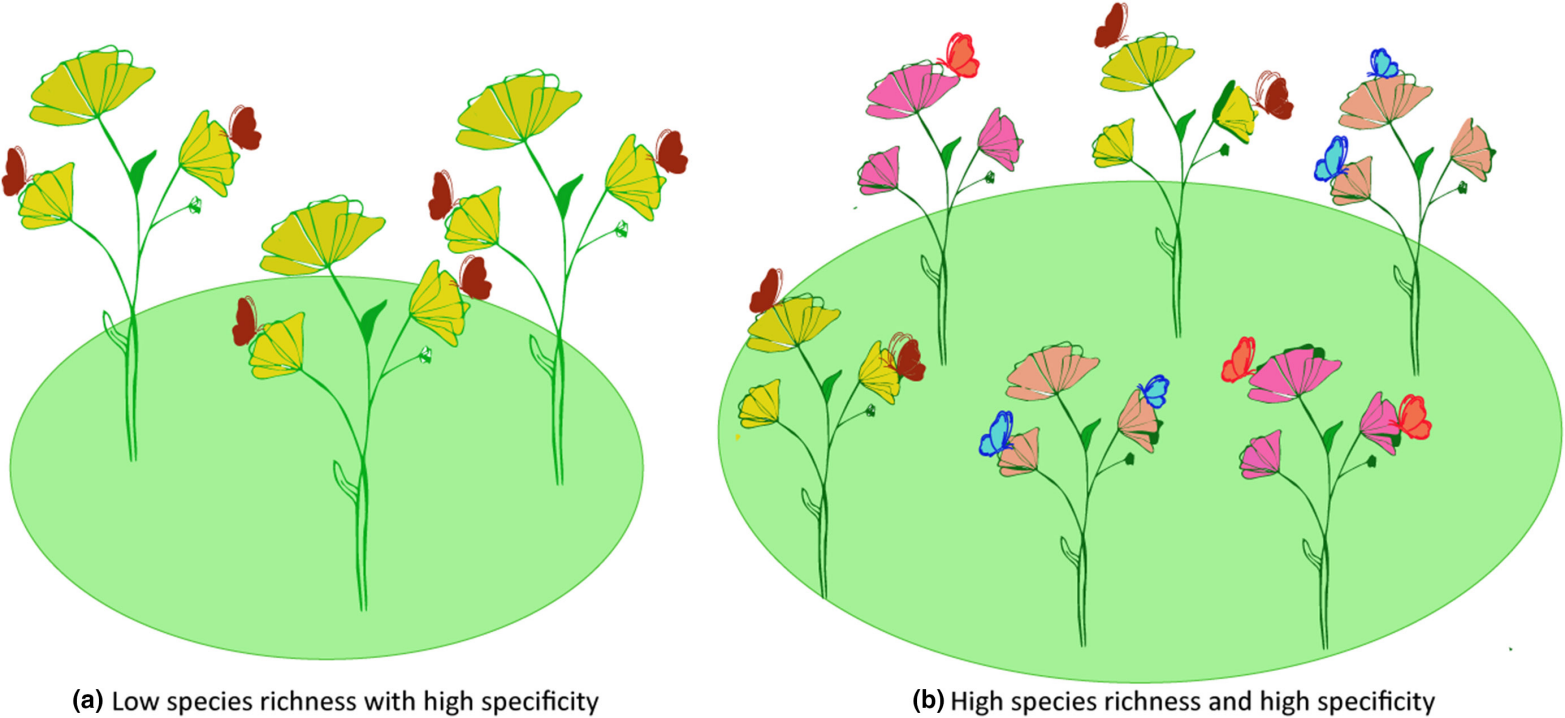
Schematic demonstrating different scales of specificity and coexistence among mutualist partner species at the local scale. (a) One‐to‐one relationship of plant and pollinator mutualist species indicative of high specificity. (b) Multiple plant and pollinator species co‐occurring, but specificity is still high at the individual level. Individuals of each plant species has its own pollinator mutualist species that is not shared with other plant species and pollinator species do not co‐occur on the same plant species.

Obligate mutualisms can form among species from across the kingdoms of life as species with complementary goods and services interact. Depending on how important those goods or services are to each partner's fitness, there may be selection to further increase the strength of the interaction and there are many examples of trait evolution in mutualistic interactions to further the mutualism (Aigner, 2004; Bronstein et al., 2006; Cook et al., 2004; Hoeksema, 2010; Pellmyr & Krenn, 2002). As part of the study of obligate mutualisms, there have been broad taxonomic and phylogenetic surveys of participating species on each side of a mutualism. In many cases, mutualist species on each side of the interaction form closely related species complexes and partner clades appear to diversify in parallel (Cruaud et al., 2012; Hayward et al., 2021; Mehdiabadi et al., 2012; Ramírez et al., 2011; Salzman et al., 2021). However, many times it appears that there are pairs of highly specific mutualist partner species that form rather than large communities of closely related species on each side of the interaction. Specificity can be assessed at multiple scales from the individual to the species level (Figure 1). For this study, we base specificity on the number of partner species that a mutualist species interacts with at a given locality, and at the individual level. There are many instances in which obligate mutualists may switch partner species across their geographic ranges, but we focused on the local scale to examine whether individuals of a mutualist species simultaneously interact with a guild of mutualist partner species and whether these interactions are stable over time. It is at this scale that direct and indirect effects among mutualist species will come into play.

We summarized partner relationships across a wide diversity of obligate mutualisms to ascertain whether obligate mutualists in general interact with one or two partner species rather than guilds of species. Although we have likely missed some obligate mutualisms, we have strived to cover the diverse taxonomic range of species engaged in obligate mutualisms to assess this pattern (Table 1). Based on the obligate mutualisms surveyed, there is a general pattern of obligate mutualists having extremely high partner specificity. Just over 50% of the obligate mutualism systems surveyed have single pairs of interacting partner species at a local scale (Table 1). If we include insects and their endosymbionts with just one other secondary endosymbiont that increases to over 80%. The exceptions to the patterns such as obligate ectomycorrhizal fungi, A.M.F. and plants, some plant‐pollinator interactions, and lichens have additional mutualist species that can coexist on the same partner individuals at some localities. The greatest exception to the pattern of high specificity is for obligately outcrossing plants that rely on animals for pollination that can have dozens of pollinator partner species. This exception suggests that one of the factors that strongly influences specificity is whether one of the partner species lives on or within its partner. In general, though, obligate mutualisms appear to have a strong trend towards a high degree of specificity of partner species. One important caveat is that quantitatively assessing this trend is difficult in that it would require determining the average number of partner species in facultative mutualisms, of which there are perhaps hundreds of systems as a comparison. In addition, the available pool of potential mutualists may be more limited in obligate mutualisms than in facultative mutualisms, perhaps driven by specialized structures to engage in the mutualism (but see below). Regardless, the very limited number of mutualist partners in many obligate mutualisms sets the stage for exploring potential explanations.

**TABLE 1:** Survey of host specificity in obligate mutualisms.

Insects and microbes			
Insect host	Intracellular symbiont	Co‐occurring symbiont species?	References
Aphids	*Buchnera aphidicola*	One secondary symbiont‐varies	Gauthier et al. (2015), Perreau et al. (2021), Xu et al. (2021)
Psyllids	*Carsonella ruddii*	One secondary symbiont‐varies	Hall et al. (2016), Nakabachi et al. (2022)
Whiteflies	*Portiera aleyrodidarum*	Secondary symbiont‐ *Hamiltonella*	Skaljac et al. (2010)
Mealybugs	*Tremblaya princeps*	Additional bacterium in *Tremblaya*	McCutcheon and von Dohlen (2011)
Phloem feeding insects	*Sulcia muelleri*	One secondary symbiont‐varies	Bennett and Moran (2013)
Curculionini weevils	*Nardonella* spp.	No	Toju et al. (2013)
*Sitophilus* weevils	*Sodalis* spp.	*Wolbachia*	Vieira and Guedes (2021)
Tsetse flies	*Wigglesworthia glossinidia*	*Sodalis glossinidius*	Dale and Welburn (2001)
Carpenter ants	*Blochmannia floridanus*	No	Zientz et al. (2006)
	**Extracellular symbiont**		
Plataspid stinkbug	*Ishikawaella capsulata*	No	Hosokawa et al. (2006)
Acanthosomatid stinkbugs	*Rosenkranzia clausaccus*	No	Kikuchi et al. (2009)
Attine ants	*Pseudonocardia*	No	Andersen et al. (2013)

	Number of partner species per individual mutualist	Additional mutualist species?	References
Insects and fungi			
Termites and fungus	1 (*Termitomyces*)	No	Mueller et al. (2005)
Ambrosia beetles	1	Secondary fungi may be present	Hulcr and Stelinski (2017)
Bark beetles	1	Secondary fungi may be present	Kandasamy et al. (2016)
Sirex wood wasps and fungus	1 (*Amylostereum*)	No	Hajek et al. (2013)
Fungi and leafcutter ants	1	No	Mueller et al. (2001)
Ants and others			
Ants and acacia	1	May include turnover of ant species	Clement et al. (2008), Palmer et al. (2003)
Ants and Lycaenids	1	No	(Pierce et al., 2002)
Ants and *Squamellaria* plants	1	3 or more species of *Squamellaria*	Chomicki et al. (2019), Ješovnik and Schultz, (2022)
Brood pollination mutualisms			
Yucca and yucca moths	1	Few instances of two	Pellmyr (2003)
Figs and fig wasps	1	Few instances of two	Cook and Segar (2010)
Leafflower and leafflower moths	Variable	Mostly 1, but 2 or 3 moth species per plant prevalent	Hembry et al. (2014)
Cycads and weevils	1	No	Salzman et al. (2021)
Cycads and thrips	1	No	Brookes et al. (2015)
Senita cactus and senita moths	1	No	Holland and Fleming (2002)
*Greya* moths and saxifrages	1	No	Davis et al. (1992)
Globeflower and globeflower flies	4 flies per globeflower	Partitioning of flower parts	Pellmyr (1989)
*Boronia* and heliozelid moths	1	No	Milla (2019)
*Bradysia* and *Rheum n*obile	1	No	Song et al. (2014)
Obligate outcrossing plants (50% of plants)	Variable	Can be dozen of pollinator species	Chomicki et al. (2020)
Plants and fungi			
A.M.F. and plants	Multiple species of fungus	Yes	Sanders (2004), van de Voorde et al. (2010)
E.C.M. and plants	4–5 E.C.M. species per tree	Yes	Smith et al. (2013)
*Epichloe* and grasses	1	No	von Cräutlein et al. (2021)
Flies and *Epichloe* spore transport	1	No	Bultman et al. (1995, 1998)
Lichens	1–3 algae species	No	Sanders and Masumoto (2021), Singh et al. (2017), Yahr et al. (2004)
Other systems			
Anemones and anemonefish	1	Some fishes use 9 anemone species	Elliott (1992)
Gobies and shrimp	1	Shrimp may use different fish	Thacker et al. (2011)
Coral and algae	1	Secondary symbiont common	Pochon and Gates (2010)
Tubeworms and endosymbionts	1	No	Gibson et al. (2010)

*Note*: Any additional mutualist partner species are indicated. Example references are provided for each system.

The striking pattern of specificity across a broad diversity of obligate mutualist systems suggests the possibility of a single process that may be responsible for keeping many obligate mutualisms close to a one‐to‐one relationship. Given the wide range of traits and services that are traded across obligate mutualisms, it seems unlikely that trait complementarity alone would lead to reciprocal specificity that would prevent other mutualists from simultaneously engaging with partner species. For example, brood pollination mutualisms with open flowers are available to other mutualists and in some localities, multiple mutualist species on each side of the interaction are in sympatry but do not share partner species (Cook & Segar, 2010; Herre et al., 2008; Pellmyr, 2003). Additionally, mutualist species have similar trait values for mutualistic traits, suggesting that they have the morphology to interact with other mutualist species should they encounter them (Althoff & Segraves, 2022). Specificity in the number of partners on each side of the interaction will be influenced by a variety of factors, including ecological context, the availability of alternative partners, and the costs and benefits of mutualism. When there are multiple species with similar traits that could potentially engage with a partner species, however, this sets the stage for competition among mutualist guild members for the partner species. Despite calls for examining the role of competition in mutualism (Johnson & Bronstein, 2019; Jones et al., 2012; Palmer et al., 2003), there is still a limited number of studies determining whether mutualistic resources and services can be a limiting resource among mutualist species and how competition among mutualists can shape mutualist communities, particularly for obligate mutualisms.

### 1.2 Q2. What experimental evidence exists for competition limiting the number of mutualist species that can coexist?

Although the study of competition has a long and rich history in ecology and evolution, there has been very limited empirical research directed at examining competition among mutualists (Palmer, 2003). Even so, there are a small number of studies demonstrating that mutualist guild members do compete and that competition can serve to limit mutualist partner species richness at a local scale. In addition, there is also evidence that other factors, such as environmental heterogeneity may be necessary to allow multiple mutualist species to interact with the same individuals and maintain mutualist partner species richness at the population level. Below we summarize some key examples of competition among mutualists for partner individuals.

There are several studies from different obligate mutualisms documenting the role of competitive hierarchies that eventually limit the number of mutualist guild members that can coexist on a single partner species. A classic example of the role of competition in determining mutualist specificity is that of some acacia‐acacia ant interactions. Single trees of the African acacia, *Acacia drepanolobium* are occupied by a single ant species even though there are four potential species (*Crematogaster sjostedti*, *C*. *mimosae*, *C*. *nigriceps*, and *Tetraponera penzigi*) that could live in and protect a tree. Ant species establishment on an individual tree is dictated by competitive hierarchies, first at the foundress level when species are trying to colonize empty trees and then at the colony level as colonies mature (Palmer et al., 2003; Stanton et al., 2002). *C. sjostedti* and *C. mimosae* excel in resource acquisition through competitive adaptations while *C. nigriceps* and *T. penzigi* are better suited for colonization of new trees. Ant diversity among individual trees in this system is maintained by colonization timing as well as changes in competitive hierarchies as colonies establish and grow. As with some acacia ants, the dinoflagellates that inhabit corals exhibit a similar pattern of shifts in specificity over time (Baker, 2003; LaJeunesse et al., 2004). During the initial colonization phase, less competitive dinoflagellate species can be established in a coral individual, but they are eventually outcompeted by a competitively dominant species. Interestingly, this pattern can be limited under low light conditions in which priority effects determine which species will remain numerically dominant within the coral, with less competitive dominant species succeeding if they get established first. A similar pattern occurs for bacteria that colonize and live in the luminescent organ of multiple squid species. The mutualistic bacterial species have a strong competitive hierarchy that limits the number of species that can live inside the organ. Nishiguchi et al. (1998) demonstrated that native strains of *Vibrio* outcompete closely related strains from other squid species when pairs of bacteria species are introduced into the light organ. They suggest that this competition helps to drive the specificity of the *Vibrio*‐squid mutualism. Taken together, these examples demonstrate how differences in competitive ability among mutualist guild members can influence local specificity and species richness.

Co‐occurring mutualist guild members may also shift their use of partner species in response to competition. Adam and Horii (2012) examined the host patterns of two *Labroides* cleaner fish species. *Labroides dimidiatu*s individuals set up cleaning stations to engage potential mutualists. In contrast, *L. bicolor* searches for potential mutualists. When the two species co‐occur, both juvenile and adult *L. bicolor* shift their use of mutualist species and focus on those not used by *L. dimidiatus*. Similarly, gobies that coexist with shrimp in their burrows also compete with one another for burrow space as only one goby can live in a shrimp burrow. Lyons (2014) examined the patterns of competitive hierarchy between an obligate mutualistic goby *Nes longus* and a facultative one, *Ctenogobius saepepallens*, by examining adult and juvenile pairings as individuals tried to gain access and use a shrimp burrow. *Nes longus* individuals were significantly better at occupying and excluding other individuals of both species from their claimed burrows. In ant‐seed dispersal mutualisms, Warren et al. (2014) demonstrated that myrmecochorous plant species in eastern North America stagger seed maturity so that smaller seeded species set earlier than larger seeded species. Ants preferentially choose large seeds and smaller seed species would have a reduced probability of dispersal if they set seed at similar times as large seeded species.

Competition among fungal mutualists and plants also plays a significant role in determining host specificity. Arbuscular mycorrhizae have been shown to compete in vitro both on the outside and within the root with more intense competition within the root, (Engelmoer et al., 2014), and laboratory studies have shown that one fungal type usually dominates within the root, although complementary fungal species in terms of plant benefits can coexist (Jansa et al., 2008). Similar patterns have been documented in ectomycorrhizal fungi in which there is strong competition for root space among E.C.M. (Ectomycorrhizal fungi) species that can lead to reduced fungal diversity on a plant (reviewed in Kennedy, 2010). Environmental context such as soil conditions, nutrient requirements of the plant and colonization dynamics can allow multiple E.C.M. species to coexist.

Perhaps the most striking example of specificity in obligate mutualisms is the species rich groups of plant pollinator brood mutualisms like figs and fig wasps and yuccas and yucca moths. In these obligate mutualisms, females use active pollination to provide resources for their developing offspring. Although there are exceptions, the general pattern in these systems is for one pollinator species to interact with one plant species at a given locality and in many cases across the plant's geographic range. In sympatry, there can be multiple pollinator‐plant pairs that do not interact with each other's mutualists. When pollinator species do coexist there are differences in key traits that suggest competition is operating. For example, Darwell and Cook (2017) demonstrate that ovipositor length of fig wasp species that use the same fig host species have shifts in egg‐laying structures that provides access to different flower types when they co‐occur in sympatry but are the same in parapatric populations. Furthermore, environmental niche modeling suggests that each fig wasp species could live in the range of the other. In China, when two fig wasp species do use the same host fig species, one species is always numerically dominant and the other occurs at a very low frequency across host plants (Yu et al., 2021). A similar pattern occurs in yuccas and yucca moths in which instances of co‐occurring pollinator moth species differ in their egg‐laying morphology with one species that deposits eggs deep in the locule of the yucca flowers and one that deposits eggs just below the pistil wall (Addicott & Tyre, 1995; Althoff, 2014). In this system, pollinators that have the same egg‐laying morphology do not co‐occur on the same yucca host individuals (Althoff, 2014). These examples from a taxonomically diverse set of interacting obligate mutualists suggest that the high degree of specificity observed in these interactions may partly be explained by competition among mutualist guild members, especially for mutualists that are smaller and live on or in structures of their mutualists.

### 1.3 Q3. What do the exceptions tell us about the potential role of competition in obligate mutualisms?

**FIGURE 2:**
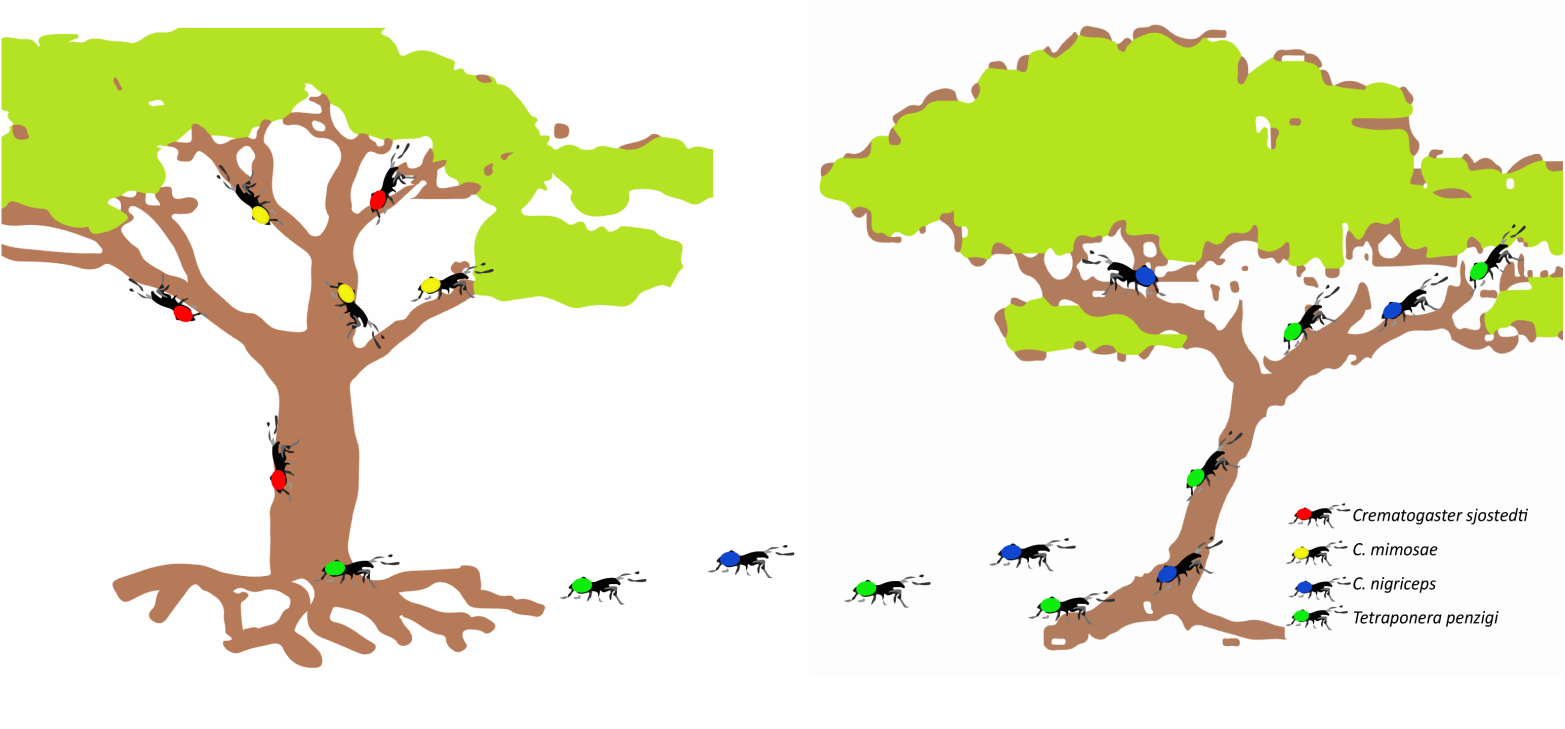
Coexistence via competition and colonization trade‐off among four species of acacia ants as elucidated by Stanton et al. (2002) and Palmer et al. (2003). *Crematogaster sjostedti* (red‐bodied ants) and *C. mimosae* (yellow‐bodied ants) are superior in displacing other species of ants whereas *C. nigriceps* (blue‐bodied ants) and *T. penzigi* (green‐bodied ants) are better colonizers of empty trees. Ant species on an individual acacia tree may cycle through ant species, but ant species coexist dynamically at the larger spatial scale across trees within a site.

As the above examples demonstrate, competition can occur and limit mutualist guild diversity. There are also instances, however, in which obligate mutualist guild members do coexist on a single partner species and this number is likely to increase as more and more studies on partner use identify the involvement of cryptic species pools (Figure 2; Machado et al., 2005) in systems thought to include one‐to‐one species interactions. Multi‐mutualist communities can also arise when there are varying mutualistic dependencies that may allow multiple mutualists to use the same partner species (Chomicki et al., 2020). For example, there is a continuum between facultative and obligate dependency that will determine how important a mutualistic interaction is for a given species, and this may drive the level of competition. Facultative mutualists have a reduced reliance on mutualistic goods or services for their fitness and in many cases can survive and reproduce without the interaction. In general, it is assumed that obligate mutualists face intense competition due to higher dependency, while facultative mutualists often can coexist due to their alternative partner species choices and resource flexibility. Thus, coexistence is more difficult for obligate mutualist guild members, especially when they live in intimate contact with their partner species.

We argue that the formation and persistence of multimutualist communities, especially in obligate mutualisms, usually involves additional mechanisms or ecological contexts that allow the coexistence of mutualist guild members. Palmer et al. (2003) reviewed these factors for mutualism in general which included niche partitioning, competition/colonization tradeoffs, environmental heterogeneity, and recruitment limitation. Each of these mechanisms has the potential to limit competition and allow the formation and persistence of multi‐mutualist communities. Resource partitioning reduces or eliminates direct competition and enhances the overall stability of the guild. The trade‐off between competition and colonization abilities and environmental heterogeneity can contribute to complex dynamics of mutualist coexistence across individuals and landscapes. Below we provide examples of each for obligate mutualisms.

**FIGURE 3:**
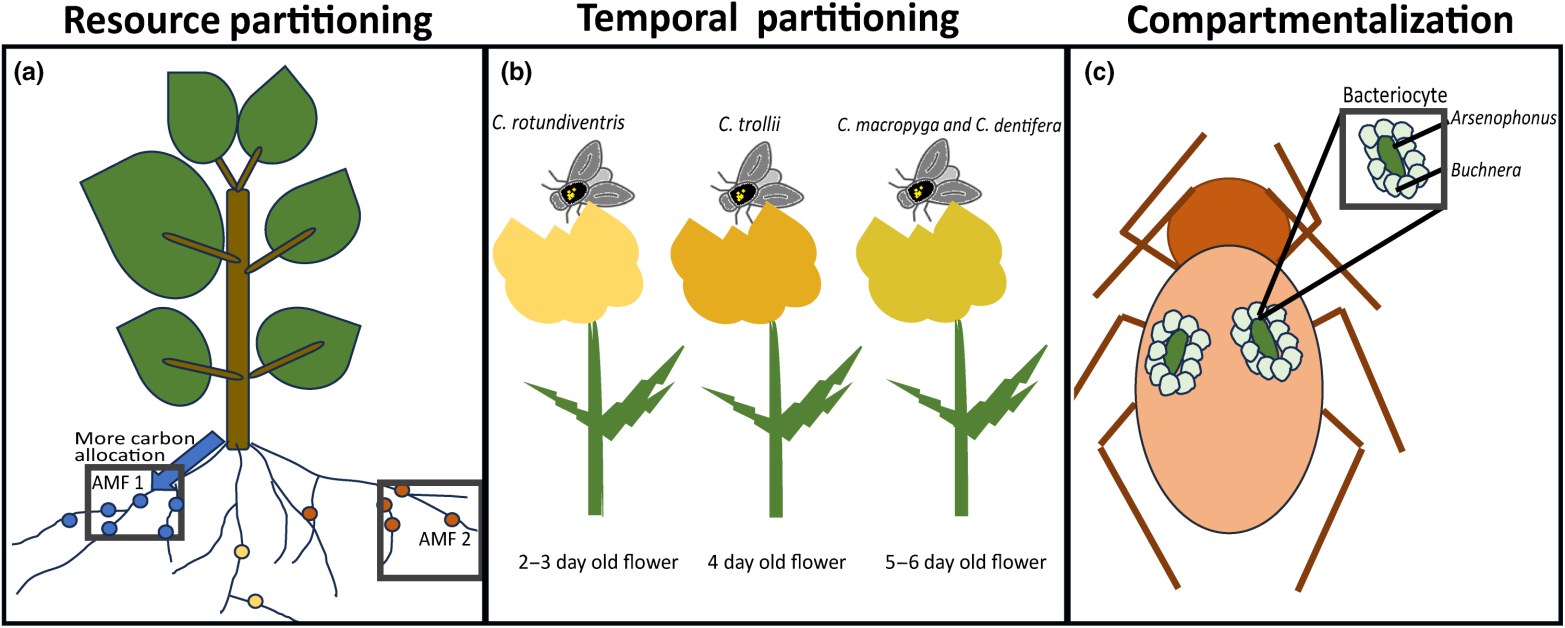
Different mechanisms of coexistence of multiple partner species in obligate mutualisms. (a) Resource partitioning by plant allowing the coexistence of multiple species of A.M.F. (b) Temporal partitioning on a single *Trollius europaeus* plant allowing the coexistence of four species of pollinating *Chiastocheta* flies. (c) Coexistence by spatial compartmentalization of two obligate symbionts, *Buchnera* and *Arsenophonus* in the aphid body.

Resource partitioning among mutualist guild members is one mechanism that can promote both ecological and evolutionary coexistence. For obligate mutualisms, however, it may be more difficult for mutualist guild members to divide up resources so that there is little overlap in mutualistic niche space. In the mutualism between insects and their endosymbionts this is accomplished via separate structures within the host to house primary endosymbionts and secondary endosymbionts (Figure 3c). These separate living spaces allows each endosymbiont to interact individually with their host insect rather than competing directly for living space and resources that may flow into that space. For mutualists that live and interact externally with their partners, some mutualists species have evolved to shift their phenologies and interact at different timepoints. An obligate pollinator guild of globeflower flies in the genus *Chiastocheta* can simultaneously use the same individuals of the globeflower plant, *Trollius europaeus*. The pollinating flies depend on the plant for pollen and nectar as well as mating and oviposition sites. Certain attributes such as a flower's longevity, blooming pattern, visual, and chemical cues facilitate the temporal partitioning (Figure 3b; Després & Cherif, 2004). Different species of flies have varying preferences for specific stages of the flower's development, such as the early, mid, or late flowering stages. This temporal variation in the flower's attractiveness allows for different species to oviposit at different times, reducing competition for larval resources (developing seeds) and increasing the overall reproductive success of the *Chiastocheta* flies (Després & Jaeger, 1999; Pellmyr, 1989; Pompanon et al., 2006). Interestingly, it is resource partitioning of larval development substrates rather than the mutualistic goods, nectar and pollen, that allows coexistence.

Coexistence within guilds also can be promoted when species trades‐off between their competitive and colonizing abilities. The species that excels at competition for mutualist partners might allocate more resources to competition or have competitive traits that reduce its ability to disperse and colonize new partners as quickly as other mutualist guild members. On the other hand, a species that focuses on colonization abilities might invest fewer resources or have traits for direct competition for partner individuals. This will create a patchwork of subpopulations in which there may be different numbers of mutualists coexisting on partner individuals as mutualist guild members turnover. This type of process operates among four species of acacia ants that compete for individuals of *Acacia drepanolobium* but manage to coexist (Stanton et al., 2002). Coexistence is made possible through a hierarchy of competitive dominance among mature ant colonies. A trade‐off occurs between highly competitive ant species within already occupied trees and the ability to colonize new trees (Figure 2). Some ant species (*Crematogaster sjostedti* and *C. mimosae*) have developed adaptations to outcompete other species in resource acquisition, giving them an advantage in acquiring key resources from the trees while other species (*C*. *nigriceps* and *Tetraponera penzigi*) have a better capability to colonize and thrive in new and unoccupied acacia trees. Thus, there are different numbers of ant species that co‐occur in individual trees and this number changes through time.

Another potential mechanism that might lead to the maintenance of multiple partners can be partner choice. In this case, the host species actively selects and favors specific mutualistic partners based on their performance or contributions, and hence promotes the coexistence of multiple mutualistic partners. Host species may evaluate the performance of mutualistic partners and preferentially allocates resources to those partners that provide the greater benefits. The mutualism among plants and A.M.F. is one such example of how partner choice may maintain mutualist guild coexistence. Plants can differentially allocate carbon resources to various E.C.M. species and create distinct resource niches within the plant roots where they preferentially interact with certain mutualistic partners over others (Figure 3a; Bachelot & Lee, 2018; Bogar et al., 2019; Hortal et al., 2017; Kiers et al., 2011). This selectivity can reduce competition among mutualists and enhance the stability of the multi‐mutualist community. Partner choice is context‐dependent and influenced by environmental conditions, resource availability, and the presence of other species (Lekberg et al., 2007).

The mutualisms discussed above suggest that competition is an important force governing species richness and the dynamics of mutualistic guilds. Additionally, results from these systems suggest that in addition to being able to function as a good partner, mutualist species must also be able to compete or curtail competition with other members of their mutualistic guild. The combination of these two factors may make it difficult for other mutualist species to invade and become established in obligate mutualisms.

## 2 FUTURE PERSPECTIVES

The extreme specificity observed in many different types of obligate mutualisms suggests that maintaining multi‐mutualist guilds that interact with a partner species is unlikely. However, various ecological, evolutionary, and environmental factors can still promote the existence of such complex networks of interactions. Historically, the evolution of mutualists to one another and the ability of mutualists to potentially choose partners have been major avenues of research in mutualism that have helped explain why and how mutualisms may be highly specific. While partner choice and the evolution of mutualistic traits between partners can drive specificity, they might not be the only explanation for the high degree of specificity in obligate mutualisms. We posit that competition among mutualist guild members may be a potent force that will serve to limit the number of partner species that can interact and drive patterns of specificity.

Using empirical studies to examine the role of competition in generating specificity in obligate mutualisms is a crucial step in understanding how mutualist communities function and are maintained. These studies will also help clarify whether the ecological and evolutionary dynamics of the mutualism are what drives specificity. The rich history of research on competition, in general, combined with studies focused on mutualistic communities, provides a strong conceptual and methodological framework for examining whether competition drives specificity in obligate mutualisms. Based on this framework, we provide a series of questions and experimental approaches for studying competition among guild members in obligate mutualisms (Table 2). Studying competition among mutualists will require an integration of studies examining a focal mutualism across its geographic range as well as experimental manipulations of mutualist community members to identify how competition or mechanisms to alleviate competition contribute to community mutualist guild structure.

**TABLE 2:** Potential tests of competitive exclusion and coexistence mechanisms at local scales for obligate mutualisms.

Pairwise mutualism and competition	
Hypothesis: Competition limits mutualism to single partner species	
Mutualist displacement (supporting pattern)	For the same mutualist species, do partner species replace each other over geographic space? examine geographic patterns of mutualist species for parapatry
Competitive dominance (experimental test)	Does “home” mutualist species outcompete “away” species when using the same partner species? perform competition trials with mutualist species on each side of the mutualism
**Mutualist guilds and coexistence** **Hypothesis: coexistence mechanisms permit multiple partner species**	
Niche partitioning (supporting pattern) (experimental test)	Do coexisting mutualist guild members use different niches on mutualistic partners? examine patterns of use in populations with and without additional guild members remove one guild member to test if others expand into its niche
Competition‐Colonization trade‐offs (supporting pattern) (experimental test)	Do mutualist guild members turnover on individuals of partner species? track guild members presence on individuals of partner species over time and examine for replacement test for competitive hierarchies among guild members

*Note*: For each potential mechanism there are patterns of species presence that support each hypothesis and experimental tests for competition.

Pairwise mutualistic interactions are perhaps the best test cases for determining the role of competition in mutualism specificity. If competition is limiting the number of mutualist species on each side of the interaction, then we would expect the replacement of mutualist species across the geographic range of its partner species which may lead to mutualist species on one side of the interaction having parapatric distributions. Competition trials among mutualist species on the same side of the interaction would provide an experimental test of whether competition is driving this pattern of replacement. Conversely, systems in which there are mutualist guilds suggest that mechanisms that allow the coexistence of competitors such as niche partitioning, colonization‐competition tradeoffs, and competitive dominance hierarchies can be examined to understand whether these mechanisms are operating. In systems such as this, perhaps the first step is to determine if mutualistic resources or services are truly limiting. Once this has been established, coupling patterns of mutualist species richness with experiments testing for mechanisms of coexistence would lead to a rigorous examination of how mutualist guild members can coexist.

## 3 CONCLUSIONS

The extreme specificity in obligate mutualisms is determined by a variety of factors and here we propose that competition may be one of the more important ones. For many systems, mutualists either live on or inside of their mutualistic partners. This generates a high degree of dependence on the partner species for fitness which will generate strong selection to defend partners and monopolize mutualistic resources. Additionally, living space may become quickly limiting causing both intra‐ and interspecific competition among mutualists. This ecological scenario will lead to competitive exclusion, and coexistence may only be possible if mechanisms that limit competition come into play. Our understanding of obligate mutualist guild structure is predicated on a very limited number of empirical studies and further empirical studies dissecting the role of competition in relation to other factors that may promote mutualist coexistence are needed. Exploring how these factors interact and influence each other will provide valuable insights into the mechanisms driving mutualist community structure.

## AUTHOR CONTRIBUTIONS

**David M. Althoff:** Conceptualization (equal); formal analysis (equal); investigation (equal); writing – original draft (equal); writing – review and editing (equal). **Renuka Agarwal:** Conceptualization (equal); formal analysis (equal); investigation (equal); writing – original draft (equal); writing – review and editing (equal).

## FUNDING INFORMATION

The research presented was supported by a grant from the U.S. National Science Foundation DEB 2137554 to D.M.A.

## Data Availability

No data were generated during this study.
